# Correcting Diagnostic Test Sensitivity and Specificity for Patient Misclassifications Resulting from Use of an Imperfect Reference Standard

**DOI:** 10.3390/diagnostics13010090

**Published:** 2022-12-28

**Authors:** Paul F. Sherwin

**Affiliations:** Clinical Development, GE HealthCare, Marlborough, MA 01752, USA; paulsherwin@ge.com

**Keywords:** imperfect standard of truth, reference standard, sensitivity, specificity

## Abstract

Investigational diagnostic tests are validated by using a reference standard (RS). If the RS is imperfect (i.e., it has sensitivity [Se] and/or specificity [Sp] < 1), incorrect values for the investigational test’s Se and Sp may result because of patient misclassification by the RS. Formulas were derived to correct a test’s Se and Sp that were determined by using an imperfect RS. The following derived formulas correct for misclassification and give the true numbers of disease-positive [n_DP_] and disease-negative patients [n_DN_] from the apparent number of disease-positive and disease-negative patients (an_DP_ and an_DN_), and the Se and Sp of the RS (Se_R_, Sp_R_): n_DP_ = (an_DP_ × Sp_R_ + an_DN_ × Sp_R_ − an_DN_)/J_R_; n_DN_ = (an_DP_ × Se_R_ + an_DN_ × Se_R_ − an_DP_)/J_R_, where J_R_ is Youden’s Index for the RS (J_R_ = Se_R_ + Sp_R_ − 1). The following derived formulas give the correct Se and Sp of an investigational test (Se_I_ and Sp_I_): Se_I_ = (an_TPI_ × Sp_R_ − n_DP_ × Se_R_ × Sp_R_ + n_DP_ × J_R_ + n_DN_ × Sp_R_^2^ − n_DN_ × Sp_R_ − Sp_R_ × an_TNI_ + an_TNI_)/(n_DP_ × J_R_); Sp_I_ = (an_TPI_ − an_TPI_ × Se_R_ + n_DP_ × Se_R_^2^ −  n_DP_ × Se_R_ − Se_R_ × n_DN_ × Sp_R_ + n_DN_ × J_R_ + Se_R_ × an_TNI_)/(n_DN_ × J_R_), where an_TPI_ is the apparent number of true-positive test results, and an_TNI_ is the apparent number of true-negative test results. The derived formulas correct for patient misclassification by an imperfect RS and give the correct values of a diagnostic test’s Se and Sp.

## 1. Introduction

Sensitivity (Se) and specificity (Sp) are fundamental measures of the performance of a diagnostic test. They, respectively, provide the probability of a positive test result in patients with the index disease (the disease that the test is designed to detect or exclude) and a negative test result in patients without the disease [1]. When combined with pre-test probability (prevalence) of disease in a tested population, they can be used to determine the more clinically useful metrics *positive predictive value* (PPV) and *negative predictive value* (NPV) [2]. Alternatively, Se and Sp can be used to determine likelihood ratios, which can be used with the pre-test odds (rather than probability) of disease to estimate the post-test odds of disease [3].

The Se and Sp of an investigational test (IT) must therefore be accurately determined before the test can be useful in clinical practice. Typically, accuracy is determined through one or more clinical studies in which study patients undergo both the IT and another diagnostic test, which serves as a reference standard (RS). The RS is used to classify each patient as disease-positive (DP) or disease-negative (DN), and these classifications are used to classify the patient’s IT result as true positive (TP), true negative (TN), false positive (FP), or false negative (FN). The respective numbers of patient classifications (n_TP_, n_TN_, n_FP_, and n_FN_) are used to determine the sensitivity and specificity of the investigational test (Se_I_ and Sp_I_, respectively; Formulas (1) and (2)).

Se_I_ = n_TP_/n_DP_ (1)

Sp_I_ = n_TN_/n_DN_ (2)

If the RS is perfect (Se_R_ = Sp_R_ = 1 [100%]), then it is a true standard of truth, and each patient is correctly classified as DP or DN. Se_I_ and Sp_I_ can then be determined straightforwardly and correctly. When a perfect RS is used to evaluate a patient, there are only two possible outcomes for the RS (either a true-positive RS result classifying the patient as DP or a true-negative RS result classifying the patient as DN); and when a perfect RS is used to classify positive and negative IT results, there can only be four possible IT classifications (TP, TN, FP, and FN).

However, if the RS is imperfect (i.e., Se_R_ and/or Sp_R_ < 1 [<100%]), then it can misclassify patients, resulting in incorrect numbers of patients with and without disease (n_DP_, n_DN_, n_TP_, and n_TN_), and thus incorrect values of Se_I_ and Sp_I_. In this case, there can be four (rather than two) possible classifications of a patient by the imperfect RS: a true-positive result classifying the patient as DP, a true-negative result classifying the patient as DN, a false-positive result misclassifying the patient as DP, or a false-negative result misclassifying the patient as DN. When the imperfect RS is used to classify the positive and negative IT results, the IT can yield true or false results, which could agree or disagree with the RS, which in turn could be true or false. As a result, there could be eight categories: TP, TN, FP, and FN from comparison of the IT results to accurate RS results, plus apparent TP, TN, FP, and FN from comparison of the IT results to inaccurate RS results. Consequently, determination of the true Se and Sp of the IT when an imperfect RS is used is not as straightforward as when the RS is perfect.

An example of an imperfect reference standard that inspired this paper was the report by McKeith and coworkers of a clinical study that determined the Se and Sp of [^123^I]ioflupane with single-photon emission computed tomography (SPECT) for assessing patients with suspected dementia with Lewy bodies (DLB) [4]. In that study, the International Consensus Criteria (ICC [5]) for diagnosing DLB were used as the RS. However, it was known from a validation study [6] that the Se and Sp of the ICC were less than perfect (0.83 and 0.95, respectively). Thus, the true Se and Sp of ioflupane for DLB were uncertain. For this reason, I sought ways to adjust the apparent values of Se and Sp of ioflupane imaging, as reported by McKeith et al. [5], to account for the known Se and Sp of the diagnostic criteria used as the reference standard.

A literature search yielded several relevant articles. Nihashi et al. [7] reported using a Bayesian latent class model for adjusting the Se and Sp of DaTscan for DLB for eight clinical studies (including follow-up data from the McKeith 2007 study [4], but not the original data), although neither the published article nor the Supplemental Materials provided sufficient details to allow one to reproduce their results.

Umemneku Chikere et al. [8] discussed three methods of correcting for the effects of an imperfect RS: Brenner [9], Gart and Buck [10], and Staquet et al. [11]. All of these authors took different approaches and reported different equations. They did not report enough detail to allow one to determine if their derivations were correct.

Trikalinos et al. mentioned the possibility of adjusting results that are based on an imperfect RS but did not report derivation of the formulas needed to do so [12].

Therefore, this work was initiated to derive formulas needed to correct for patient misclassifications by an imperfect RS and to determine the true values of a diagnostic test’s Se and Sp when they were determined by using an imperfect RS, in diagnostic terms readily understandable to clinicians, with full transparency of the derivations. Those results are reported here; application of the results to the McKeith ioflupane study [4] along with a review of relevant literature, will be reported separately.

## 2. Materials and Methods

Formulas were derived on the basis of an analysis of how a reference standard (RS) is used to classify patients as disease-positive or disease-negative and how misclassifications by an imperfect RS affect the apparent values of Se_I_ and Sp_I_. Throughout, conditional independence of the RS and IT is assumed; i.e., the RS and IT misclassify patients independently. This assumption is reasonable if, for example, the RS and IT work by different mechanisms.

**Figure 1:**
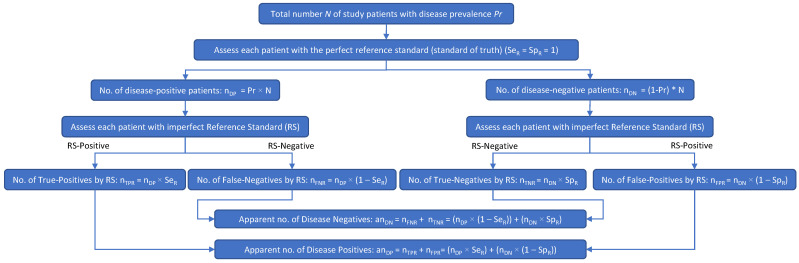
When an imperfect RS is used in a clinical study of an investigational test, the apparent number of disease-positive subjects may include some subjects who are actually disease negative, as well as subjects who are truly disease positive. Likewise, the apparent number of disease-negative subjects may include some subjects who are actually disease positive, as well as subjects who are truly disease negative.

**Figure 2:**
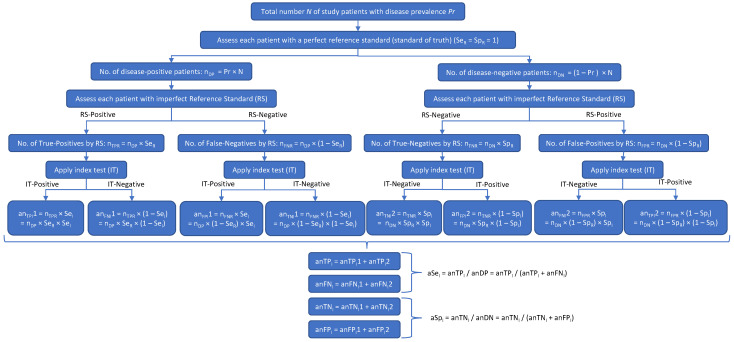
A disease may be present (DP) or absent (DN) in a subject. The result of the reference standard (RS) test may be accurate (true positive [TPR] or true negative [TNR]) or inaccurate (false positive [FPR] or false negative [FNR]). The result of the investigational test (IT) may then agree with the RS (yielding an apparent number of true positives [an_TP_] and an apparent number of true negatives [an_TN_]) or disagree with the RS (yielding an apparent number of false positives [an_FP_] and an apparent number of false negatives [an_FN_]). The an_TP_ will equal the number of subjects who got a true-positive result on both the RS and the IT, plus the number of subjects who got a false-positive result on both the RS and the IT. Likewise, an_TN_ will equal the number of subjects with a true-negative result on both the RS and the IT, plus the number of subjects with a false-negative result on both the RS and the IT. Thus, both the apparent sensitivity and the apparent specificity of the IT (i.e., the sensitivity and specificity calculated in the clinical study) will depend on both the sensitivity and specificity of the RS (Se_R_ and Se_R_).

Two diagrams were created to depict patient misclassifications by the RS (Figure 1) and their effect on the apparent values of Se_I_ and Sp_I_ (Figure 2). In both figures, the prefix *a* was used to denote an *apparent* value. In Figure 2, to differentiate between the reference and investigational tests with respect to the numbers of true positives (n_TP_), true negatives (n_TN_), false positives (n_FP_), false negatives (n_FN_), Se, and Sp, these variables had the subscripts *R* (for *reference* test) or *I* (for *investigational* test) added.

Figure 1 shows how an imperfect RS results in patient misclassifications: multiplying Se_R_ and Sp_R_ by the true numbers of disease-positive (n_DP_) and disease-negative (n_DN_) patients results in the apparent number of disease-positive patients (an_DP_) and the apparent number of disease-negative patients (an_DN_). In Figure 1, n_TPR_ is the number of patients with true-positive RS results, n_FNR_ is the number of patients with false-negative RS results, n_TNR_ is the number of patients with true-negative RS results, n_FPR_ is the number of patients with false-positive RS results, Se_R_ is the sensitivity of the reference standard, Sp_R_ is the specificity of the reference standard, an_DP_ is the apparent number of disease-positive patients, and an_DN_ is the apparent number of disease-negative patients.

Figure 2 shows how the patient misclassifications result in incorrect values of the IT’s sensitivity (Se_I_) and specificity (Sp_I_): multiplying an_DP_ and an_DN_ by the true (but initially unknown) values of Se_I_ and Sp_I_ gives the apparent numbers of true-positive (an_TPI_) and true-negative (an_TNI_) IT results, which, when, respectively, divided by an_DP_ and an_DN_ (in accordance with Equations (1) and (2) above), give incorrect apparent values of the IT’s Se and Sp (aSe_I_ and aSp_I_). In Figure 2,

- n_DP_ is the true number of disease-positive patients
- n_DN_ is the true number of disease-negative patients
- n_TPR_ is the number of patients with true-positive RS results
- n_FNR_ is the number of patients with false-negative RS results
- n_TNR_ is the number of patients with true-negative RS results
- n_FPR_ is the number of patients with false-positive RS results
- Se_R_ is the sensitivity of the RS
- Sp_R_ is the specificity of the RS
- an_DP_ is the apparent number of disease-positive patients according to the RS
- an_DN_ is the apparent number of disease-negative patients according to the RS
- Se_I_ is the sensitivity of the investigational test
- Sp_I_ is the specificity of the investigational test
- an_TPI_1 is the apparent number of true-positive IT results based on n_TPR_
- an_TPI_2 is the apparent number of true-positive IT results based on n_FPR_
- an_FNI_1 is the apparent number of false-negative IT results based on n_TPR_
- an_FNI_2 is the apparent number of false-negative IT results based on n_FPR_
- an_FPI_1 is the apparent number of false-positive IT results based on n_FNR_
- an_FPI_2 is the apparent number of false-positive IT results based on n_TNR_
- an_TNI_1 is the apparent number of true-negative IT results based on n_FNR_
- an_TNI_2 is the apparent number of true-negative IT results based on n_TNR_
- an_TPI_ is the apparent total number of true-positive IT results
- an_TNI_ is the apparent total number of true-negative IT results.

The two diagrams were analyzed to develop formulas that were then solved to give n_DP_, n_DN_, Se_I_ and Sp_I_ starting from the apparent results of a clinical study.

## 3. Results

### 3.1. Correction for Patient Misclassifications by an Imperfect RS: Calculation of True Numbers of Disease-Positive and Disease-Negative Patients

Figure 1 shows the relationship between n_DP_, n_DN_, Se_R_, Sp_R_, an_DP_, and an_DN_. From Figure 1, Formulas (3) and (4) can be deduced:

an_DP_ = n_DP_ × Se_R_ + n_DN_ × (1 − Sp_R_) (3)

an_DN_ = n_DP_ × (1 − Se_R_) + n_DN_ × Sp_R_ (4)

If an_DP_, an_DN_, Se_R_, and Sp_R_ are all known, then Formulas (3) and (4) constitute a system of equations with two unknowns (n_DP_ and n_DN_). This was solved by using an online system-of-equations calculator [13]. However, it was first necessary to substitute single-letter variables (e.g., *x* for n_DP_, *y* for n_DN_, and *a*, *b*, *c*, etc. for the other variables), because the online calculator interpreted two-letter variables as two variables rather than as a single variable. Solution of the system of equations leads to Formulas (5) and (6) (details shown in Supplementary Materials).

n_DP_ = (an_DP_ × Sp_R_ + Sp_R_ × an_DN_ − an_DN_)/(Se_R_ + Sp_R_ − 1) (5)

n_DN_ = (an_DP_ × Se_R_ − an_DP_ + Se_R_ × an_DN_)/(Se_R_ + Sp_R_ − 1) (6)

The denominators in Formulas (5) and (6) are equal to Youden’s J statistic [14] (Youden’s Index) for the RS (Equation (7)):

J_R_ = Se_R_ + Sp_R_ − 1 (7)

Therefore, Formulas (5) and (6) can be rewritten as Formulas (8) and (9):

n_DP_ = (an_DP_ × Sp_R_ + Sp_R_ × an_DN_ − an_DN_)/J_R_ (8)

n_DN_ = (an_DP_ × Se_R_ + Se_R_ × an_DN_ − an_DP_)/J_R_ (9)

Note that because the sum of n_DP_ and n_DN_ must equal N (the number of patients in the study; Equation (10)), one could also calculate just one value (either n_DP_ or n_DN_), and subtract it from N to get the other value (Equation (11) or Equation (12)):

N = n_DP_ + n_DN_ (10)

n_DP_ = N − n_DN_ (11)

n_DN_ = N − n_DP_ (12)

### 3.2. Calculation of Sensitivity and Specificity of the Investigational Test

Next, expressions for the true numbers of patients with true-positive and true-negative investigational test results (n_TPI_ and n_TNI_, respectively) were derived. Figure 2 shows the possible outcomes of a clinical study of an IT using a RS. From Figure 2, it can be deduced that:

an_TPI_ = n_DP_ × Se_R_ × Se_I_ + n_DN_ × (1 − Sp_R_) × (1 − Sp_I_) (13)

an_TNI_ = n_DP_ × (1 − Se_R_) × (1 − Se_I_) + n_DN_ × Sp_R_ × Sp_I_ (14)

If n_DP_, n_DN_, Se_R_, and Sp_R_ are all known, then Equations (13) and (14) constitute a system of equations with two unknowns (Se_I_ and Sp_I_). Substituting single-letter variables and using the same online system-of-equations calculator referenced above leads to Equation (15) for the sensitivity of the investigational test (Se_I_) and Equation (16) for the specificity of the investigational test (Sp_I_).

Se_I_ = (an_TPI_ × Sp_R_ − n_DP_ × Se_R_ × Sp_R_ + n_DP_ × Se_R_ + n_DP_ × Sp_R_ − n_DP_ + n_DN_ × Sp_R_^2^ − n_DN_ × Sp_R_ − Sp_R_ × an_TNI_ + an_TNI_)/(n_DP_ × (Se_R_ + Sp_R_ − 1)) (15)

Sp_I_ = (−an_TPI_ × Se_R _ + an_TPI_ + n_DP_ × Se_R_^2^ − n_DP_ × Se_R_ − Se_R_ × n_DN_ × Sp_R_ + Se_R_ × n_DN_ + Se_R_ × an_TNI_ + n_DN_ × Sp_R_ − n_DN_)/(n_DN_ × (Se_R_ + Sp_R_ − 1)) (16)

These can be simplified somewhat by substituting some of the terms with Youden’s Index. Thus, Equation (15) becomes Equation (17):

Se_I_ = (an_TPI_ × Sp_R_ − n_DP_ × Se_R_ × Sp_R_ + n_DP_ × J_R_ + n_DN_ × Sp_R_^2^ − n_DN_ × Sp_R_ − Sp_R_ × an_TNI_ + an_TNI_)/(n_DP_ × J_R_) (17)

Similarly, substituting some terms with Youden’s Index converts Equation (16) into Equation (18):

Sp_I_ = (−an_TPI_ × Se_R_ + an_TPI_ + n_DP_ × Se_R_^2^ − n_DP_ × Se_R_ − Se_R_ × n_DN_ × Sp_R_ + n_DN_ × J_R_ + Se_R_ × an_TNI_)/(n_DN_ × J_R_) (18)

As a quick check of the validity of the equations, if Se_R_ = Sp_R_ = 1, then Equations (17) and (18) should simplify to show that Se_I_ equals an_TPI_/n_DP_ and that Sp_I_ equals an_TNI_/n_DN_—and they do (See Supplementary Materials).

### 3.3. Calculation of Apparent Sensitivity and Specificity of the Investigational Test

For the sake of completeness, Equations (19) and (20), which show the relationship between n_DP_, n_DN_, Se_R_, Sp_R_, Se_I_, and Sp_I_ and the *apparent* sensitivity (aSe_I_) and specificity (aSp_I_) of the investigational test were deduced from Figure 2. Details are provided in the Supplementary Materials.

aSe_I_ = (n_DP_ × Se_R_ × Se_I_ + n_DN_ × (1 − Sp_R_) × (1 − Sp_I_))/(n_DP_ × Se_R_ + n_DN_ × (1 − Sp_R_)) (19)

aSp_I_ = (n_DP_ × (1 − Se_R_) × (1 − Se_I_) + n_DN_ × Sp_R_ × Sp_I_)/(n_DP_ × (1 − Se_R_) + (n_DN_ × Sp_R_)) (20)

### 3.4. Proportion-Based Equations

The above equations are in terms of patient counts. They can be converted to equations that are based on proportions by dividing by N, the total number of patients in the study. For example, starting with Equation (17) and dividing every term by N (equivalent to multiplying both the numerator and denominator by 1/N), one gets Equation (21):

Se_I_ = ((an_TPI_ × Sp_R_/N) − (n_DP_ × Se_R_ × Sp_R_/N) + (n_DP_ × J_R_/N) + (n_DN_ × Sp_R_^2^/N) − (n_DN_ × Sp_R_/N) − (Sp_R_ × an_TNI_/N) + (an_TNI_/N))/(n_DP_ × J_R_/N) (21)

Since n_DP_/N = Pr (the prevalence of the index disease), and since n_DN_/N = 1 − Pr, Equation (21) can be rewritten as Equation (22):

Se_I_ = (pa_TPI_ × Sp_R_ − Pr × Se_R_ × Sp_R_ + Pr × J_R_ + (1 − Pr) × Sp_R_^2^ − (1 − Pr) × Sp_R_ − Sp_R_ × pa_TNI_ + pa_TNI_)/(Pr × J_R_) (22)

In Equation (22), pa_TNI_ is the proportion of patients with an apparently true-negative investigational test result, Pr is the prevalence of the index disease, J_R_ is Youden’s Index for the RS, pa_TPI_ is the proportion of patients with an apparently true-positive investigational test result, Sp_R_ is the specificity of the RS, and Se_R_ is the sensitivity of the RS.

Similarly, dividing every term in Equation (18) by N gives Equation (23):

Sp_I_ = (pa_TPI_ − pa_TPI_ × Se_R_ + Pr × Se_R_^2^ − Pr × Se_R_ − Se_R_ × (1 − Pr) × Sp_R_ + (1 − Pr) × J_R_ + Se_R_ × pa_TNI_)/((1 − Pr) × J_R_) (23)

In Equation (23), the variables have the same meaning as in Equation (22).

### 3.5. Example Calculation Using Test Data

As an example, suppose a clinical study of an investigational diagnostic test using an imperfect RS finds that 50 patients are apparently disease-positive by the RS (an_DP_ = 50), and 60 are apparently disease-negative (an_DN_ = 60). Suppose that Se_R_ = 0.90 and Sp_R_ = 0.85, and that the numbers of patients with apparently true IT classifications (an_TPI_ and an_TNI_) are, respectively, 38 and 48. These data correspond to aSe_I_ = 0.76 and aSp_I_ = 0.80. Youden’s Index (J_R_) for the reference standard = 0.75. Using Formula (8), n_DP_ = 45 after rounding. Since N = n_DP_ + n_DN_, then n_DN_ = 110 − 45, or 65. The number of patients misclassified by the RS is the absolute value of the difference between an_DP_ and n_DP_ (or an_DN_ and n_DN_):

Misclassifications = |an_DP_ − n_DP_| = |an_DN_ − n_DN_| = |50 − 45| = |60 − 65| = 5

Se_I_ and Sp_I_ are found by using Formulas (17) and (18): Se_I_ = 0.91 and Sp_I_ = 0.86. In this example, adjustment for the Se_R_ and Sp_R_ (0.90 and 0.85, respectively) of the imperfect RS led to an increase from aSe_I_ = 0.76 to Se_I_ = 0.91 and an increase from aSp_I_ = 0.80 to Sp_I_ = 0.86.

Suppose that in the above example Se_R_ equaled 0.75 and Sp_R_ equaled 0.90, and all of the other numbers were the same. Using Formula (8), n_DP_ = 60 after rounding. Since N = n_DP_ + n_DN_, then n_DN_ = 110 − 60, or 50. The number of patients misclassified by the RS is then 10. Se_I_ and Sp_I_ are found by using Formulas (17) and (18): Se_I_ = 0.85 and Sp_I_ = 1.00. In this example, adjustment for the Se_R_ and Sp_R_ (0.75 and 0.90, respectively) of the imperfect RS led to an increase from aSe_I_ = 0.76 to Se_I_ = 0.85 and an increase from aSp_I_ = 0.80 to Sp_I_ = 1.00.

## 4. Discussion

In assessing an investigational new diagnostic test, it is not always feasible to use a perfect RS, and an imperfect RS (one with Se and/or Sp < 1) must sometimes be used, which can result in patient misclassifications and incorrect values of Se_I_ and Sp_I_. Such situations raise questions about the accuracy of the investigational test’s Se and Sp determined using the imperfect RS. In this work, formulas for correctly calculating the investigational test’s true Se and true Sp from any reference standard were derived.

Three prior studies [9,10,11] reported derivations of formulas for correcting Se and Sp for misclassification by an imperfect RS. Their approaches differed from each other and from the approach taken here. In addition, they did not report enough detail to allow one to determine if their derivations were correct. Therefore, comparison of this work to theirs was difficult but was successful for the approaches by Gart and Buck [10] and Staquet et al. [11] (See Supplementary Materials for details).

Brenner [9] reported equations for aSe_I_ and aSp_I_ for a case–control study if Se_I_, Sp_I_, Se_R_, Sp_R_ and the exposure Pr were all known, but did not report solving for Se_I_ and Sp_I_, contrary to the paper by Umemneku Chikere et al. [8] who I believe may have misinterpreted the Brenner equations for aSe_I_ and aSp_I_ as being for Se_I_ and Sp_I_.

Gart and Buck [10] discussed the use of screening and reference tests for estimating disease Pr in epidemiologic studies. They derived equations for what they termed co-positivity and co-negativity (which I determined to be equivalent to aSe_I_ and aSp_I_), and solved these for Se_I_ and Sp_I_ if Pr, Se_R_ and Sp_R_, aSe_I_ and aSp_I_ are known. I was able to show that my equations for Se_I_ and Sp_I_ (after transformation into proportion-based variables) were equivalent to theirs (see Supplementary Materials for details).

Staquet and colleagues [11] reported equations for calculating Se_I_ and Sp_I_ provided that Se_R_, Sp_R_, and an_TPI_, an_TNI_, an_FPI_, and an_FNI_ are known. I was able to show that my equations for Se_I_ and Sp_I_ were equivalent to theirs (see Supplementary Materials for details).

Trikalinos et al. [12] did not report equations for Se_I_ or Sp_I_, but I compared their equations for the cells of their 2 × 2 contingency table (corresponding to pa_TPI_ and pa_TNI_), and they matched what I had derived (data not shown).

### Strengths and Weaknesses of This Work

This work builds on that of Trikalinos et al. [12], Gart and Buck [10], Staquet et al. [11] and Brenner [9]. These authors discussed potential methods of handling diagnostic studies that use an imperfect RS but did not provide sufficient detail to allow easy replication of their methods. Although Trikalinos et al. [12] mentioned the possibility of adjusting results that are based on an imperfect RS, they did not report derivation of the formulas needed to do so, as I have. One advantage of my work is that I provide full details of the derivations (see Supplementary Materials) so that others may easily reproduce and confirm my work. In addition, I report formulas that can use either absolute patient counts or proportions (e.g., prevalence), in contrast to prior authors, who reported formulas for only one or the other approach.

## 5. Conclusions

Validation of a new diagnostic test by use of an imperfect RS (one with Se and/or Sp < 1) introduces patient misclassifications that result in deviation of the apparent sensitivity and specificity of the index test (aSe_I_ and aSp_I_, respectively) from the true values. By analyzing the role of the reference standard in the determination of the sensitivity and specificity of an index test, it is possible to derive formulas that correct for patient misclassification by an imperfect RS, as well as for the subsequent error introduced into aSe_I_ and aSp_I_. The analysis showed that the more imperfect the RS (i.e., the lower the Se and Sp of the RS), the greater the error introduced into aSe_I_ and aSp_I_. Therefore, when an imperfect RS is used to validate a diagnostic test, it may be necessary to apply corrections to arrive at accurate values of Se and Sp for the test.

This work builds on that of prior authors who discussed potential methods of handling diagnostic studies that use an imperfect RS but did not provide sufficient detail to allow easy replication of their methods. In contrast, full details of the derivations are provided (in Supplementary Materials) to provide transparency, so that others may confirm and perhaps build upon this work. In addition, formulas based on both patient counts and patient proportions are provided, in contrast to prior authors, who provided either one or the other.

For this corrective method to be feasible, two conditions must be met. First, the assumption of conditional independence of the index test and the RS must be true; this assumption is reasonable if the two tests work by different mechanisms (e.g., if the index test relies on laboratory methods and the RS relies on autopsy). Second, one obviously needs to know the values of the Se and Sp of the RS, which may not always be the case. However, if they are known, then the derived formulas can help provide needed corrections to the apparent values of an index test’s Se and Sp.

## Data Availability

No data were collected in this research. Full derivations of equations are available in the Supplementary Materials.

## Supplementary Materials

The following supporting information can be downloaded at: https://www.mdpi.com/article/10.3390/diagnostics13010090/s1. Reference [15] is cited in the supplementary materials.
