# Supplementary material for: Correcting Diagnostic Test Sensitivity and Specificity for Patient Misclassifications Resulting from Use of an Imperfect Reference Standard
Source: Diagnostics (Basel). 2022 Dec 28;13(1):90. doi: 10.3390/diagnostics13010090 (PMC9818831; doi:10.3390/diagnostics13010090)
Supplement: Supplementary file 1 [file diagnostics-13-00090-s001.zip › diagnostics-2102461-supplementary.pdf]

Correcting Diagnostic Test Sensitivity and Specificity for Patient Misclassifications Resulting From Use of an Imperfect Reference Standard: Supplementary Material

Paul F. Sherwin, MD, PhD[1*]

[1] Clinical Development, GE HealthCare, 01752 Marlborough, MA, USA; paulsherwin@ge.com

1. Background and Objectives

An investigational diagnostic test (IT) for detecting or excluding a specific disease is usually validated in a clinical study in which the test is administered to subjects, each of whom has a known disease status (disease-positive [DP] or disease-negative [DN]). For each subject, the results of the IT, whether positive or negative, are classified as *true* if they agree with the subject's disease status (positive or negative) and false if they disagree with the subject's disease status (positive or negative). Thus, the result of the IT for each subject is classified as true positive (TP), false negative (FN), true negative (TN), or false positive (FP).

As Equation (S1) shows, the sensitivity of the IT (Se_I) is equal to the number of subjects with TN results from the IT (n_{TPI}) divided by the number of patients who are disease positive (n_{DP}) .

$$Se_I = n_{TPI} / n_{DP} \tag{S1}$$

As Equation (S2) shows, the specificity of the IT (Sp_I) is equal to the number of patients with true-negative results from the IT (n_{TNI}) divided by the number of subjects who are disease negative (n_{DN}).

$$Sp_I = n_{TNI} / n_{DN} \tag{S2}$$

Yet to calculate Se_I and Sp_I, one needs a reference standard (RS)—some independent way to establish each subject's disease status. The RS is generally another diagnostic test that is assumed to be perfect, i.e., to give the true disease status in 100% of patients. If the RS is a true standard of truth or gold standard, then its sensitivity (Se_R) and specificity (Sp_R) will both be 1 (100%). If Se_R is 1, then all of the subjects with positive results from the RS will truly have the disease. As a result, the number of true-positive results from the RS (n_{TPR}) will equal the number of disease-positive subjects (n_{DP}). Likewise, if Sp_R is 1, then everyone with a negative result from the RS will be truly free of the disease. Thus, the number of true-negative results from the RS (n_{TNR}) will equal the number of disease-negative subjects (n_{DN}). If Se_R and Sp_R are 100%, then it is easy to validate the IT (Equations (S1) and (S2), and Figure S1).

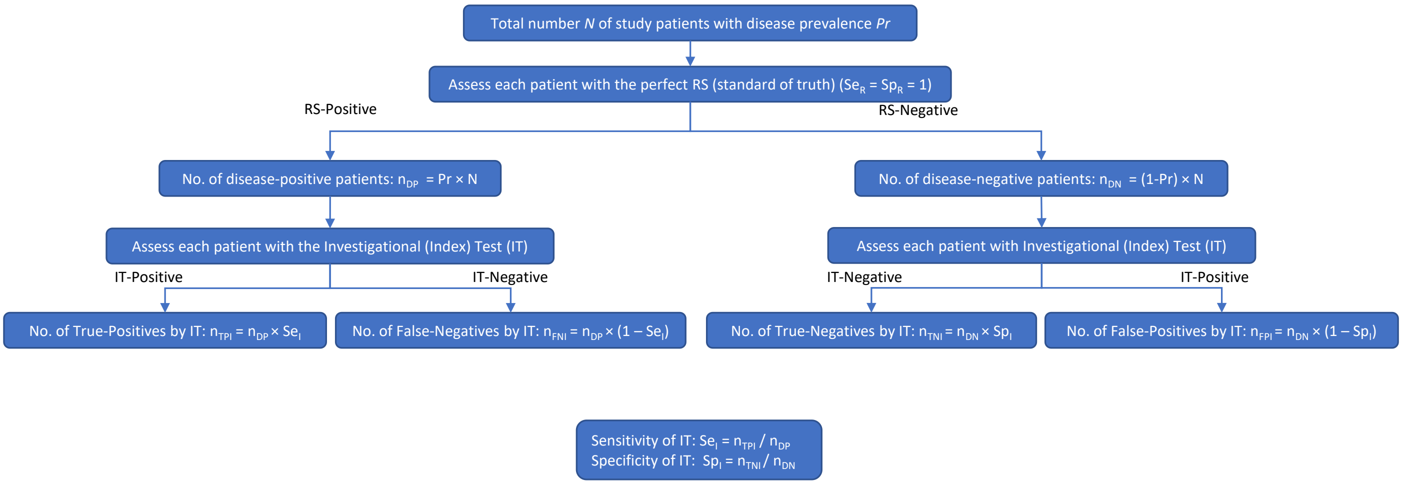

Figure S1. If a reference standard (RS) is perfect, its sensitivity [Se_R] and specificity [Sp_R] are both equal to 1. All of the subjects with a positive RS result will be disease-positive, and all disease-positive patients will have a positive RS result. Thus, the number of subjects with a positive RS result can be used as the denominator for calculating sensitivity of the investigational test (Se_I). Likewise, all subjects with a negative RS result will be disease negative, and all disease-negative subjects will have a negative RS result. Thus, the number of subjects with a negative RS result can be used as the denominator for calculating the specificity of the investigational test (Sp_I).

Unfortunately, if the RS is imperfect (Se_R and/or $Sp_R < 1$), then the RS may misclassify patients. As a result, Figure S1 and Equations (S1) and (S2) would no longer apply. To determine the correct values of Se_I and Sp_I, one must adjust for the misclassification due to the imperfect RS.

The objective of this work was to derive formulas to correct for patient misclassification by an imperfect RS as well as to correct the error that patient misclassification introduces into the determination of an IT's sensitivity (Se_I) and specificity (Sp_I). The derivations using patient counts were done first. The resulting formulas were then converted into formulas based on patient proportions.

2. Derivations Using Patient Counts

2.1 Numbers of Disease-Positive and Disease-Negative Patients

If the RS is imperfect (Se_R and/or Sp_R <1), then use of the RS could lead to some false results (FN_R and FP_R), as well as true results (TP_R and TN_R). The subjects with an FN_R or FP_R result would thus be misclassified with respect to the disease status of interest. The comparison of the results of the IT to the results of an imperfect RS yields apparent results (positive or negative) that could be true or false (the prefix a is used to indicate apparent): aTN, aTP, aFN, aFP. Figure S2 shows how the *apparent* numbers of disease-negative patients (an_{DN}) and disease-positive patients (an_{DN}) that are observed in a clinical trial depend on Se_R, Sp_R, n_{DP}, and n_{DN}.

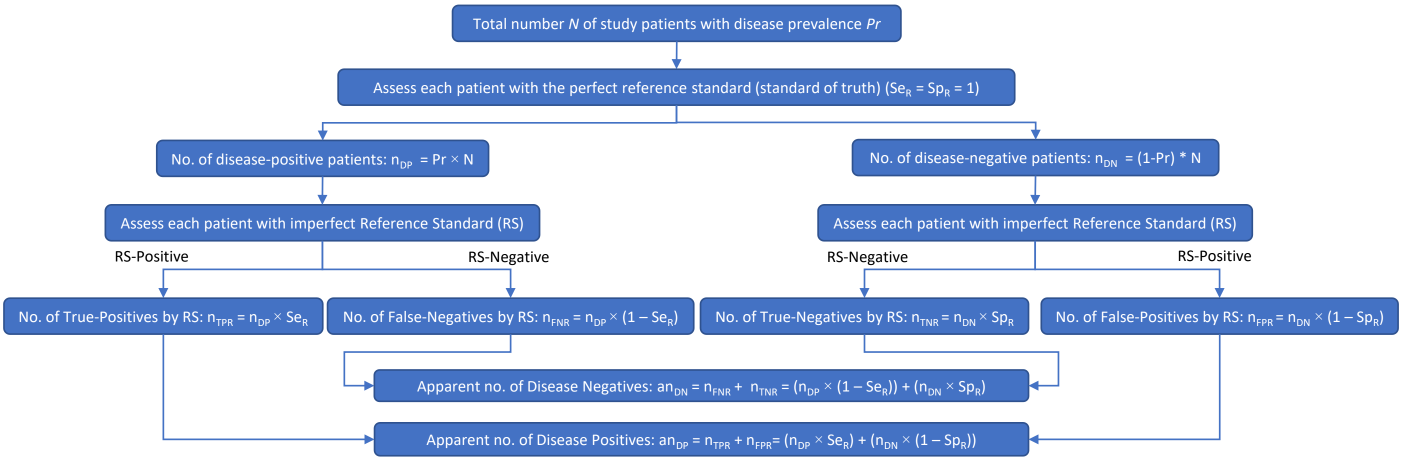

Figure S2. If the RS used in a study of an IT is imperfect (i.e., the Se_R and/or Sp_R <1), then some of the subjects may be misclassified as to their disease status. As a result, the apparent number of disease-positive subjects (an_{DP}) may not equal the true number of disease-positive subjects (n_{DP}). Likewise, an_{DN} may not equal n_{DN}. These discrepancies would lead to inaccuracy in the estimation of the IT's sensitivity and specificity (Se_I and Sp_I).

In Figure S2, variables are defined as follows:

- n_{DP} is the number of disease-positive patients
- n_{DN} is the number of disease-negative patients
- n_{TPR} is the number of patients with true-positive reference standard (RS) results
- n_{FNR} is the number of patients with false-negative RS results
- n_{TNR} is the number of patients with true-negative RS results
- n_{FPR} is the number of patients with false-positive RS results
- Se_R is the sensitivity of the RS
- Sp_R is the specificity of the RS
- an_{DP} is the apparent number of disease-positive patients according to the imperfect RS
- an_{DN} is the apparent number of disease-negative patients according to the imperfect RS

Based on Figure S2, formulas (S3) and (S4) (reported as formulas 3 and 4 in the manuscript) can be deduced:

$$an_{DP} = n_{DP} \times Se_R + n_{DN} \times (1 - Sp_R) \quad \text{(S3)}$$

$$an_{DN} = n_{DP} \times (1 - Se_R) + n_{DN} \times Sp_R \quad \text{(S4)}$$

Note that if the RS is perfect (i.e., $Se_R = Sp_R = 1$), then none of the subjects would be misclassified, so Equation (S3) will reduce to $an_{DP} = n_{DP}$ and Equation (S4) will reduce to $an_{DN} = n_{DN}$.

If an_{DP}, an_{DN}, Se_R, and Sp_R are all known, then Equations (S3) and (S4) constitute a system of equations with 2 unknowns: n_{DP} and n_{DN}. Although this can be solved manually, online calculators are available which can solve systems of equations much faster. The calculators I tried could not handle variables with more than 1 letter, so single-letter variables were substituted into Equations (S3) and (S4). The single-letter variable substitutions were: $n_{DP} = x$ and $n_{DN} = y$, $an_{DP} = a$, $Se_R = b$, $Sp_R = c$, and $an_{DN} = d$. Rewriting the equations with the new variables, formula (S3) becomes (S5):

$$a = x \times b + y \times (1 - c) \quad \text{(S5)}$$

Similarly, formula (S4) becomes (S6):

$$d = x \times (1 - b) + y \times c \quad \text{(S6)}$$

This system of equations $a = x \times b + y \times (1 - c)$, $d = x \times (1 - b) + y \times c$ was entered into an online calculator [1] to solve for x and y. The solutions were (S7) and (S8):

$$x = (a \times c + c \times d - d)/(b + c - 1) \quad \text{(S7)}$$

$$y = (a \times b - a + b \times d)/(b + \mathrm{c} - 1) \quad \text{(S8)}$$

Resubstituting the original variables gives formulas (S9) and (S10), which are equivalent to formulas 5 and 6 in the manuscript:

$$n_{DP} = (an_{DP} \times Sp_R + Sp_R \times an_{DN} - an_{DN}) \;/\; (Se_R + Sp_R - 1) \quad \text{(S9)}$$

$$n_{DN} = (an_{DP} \times Se_R - an_{DP} + Se_R \times an_{DN}) \;/\; (Se_R + Sp_R - 1) \quad \text{(S10)}$$

The denominators in (S9) and (S10) each equal Youden's J statistic [2] for the RS ($J_R = Se_R + Sp_R - 1$), which can be used to simplify derivations of other equations (see below).

2.2 *Investigational Test Sensitivity and Specificity (Se_I and Sp_I)*

From Equations (S1) and (S2), it can be appreciated that the *apparent* values of Se and Sp for the IT (aSe_I and aSp_I) that are observed in a clinical study that uses an imperfect RS are given by the apparent number of true-positive IT results (an_{TPI}), the apparent number of disease-positive patients (an_{DP}), the apparent number of true-negative IT results (an_{TNI}), and the apparent number of disease-negative patients (an_{DN}). I just showed how an_{DP} and an_{DN} depend on Se_R and Sp_R. Now I will show how an_{TPI} and an_{TNI} depend on n_{DP}, n_{DN}, Se_R, Sp_R, Se_I and Sp_I. This is depicted in Figure S3.

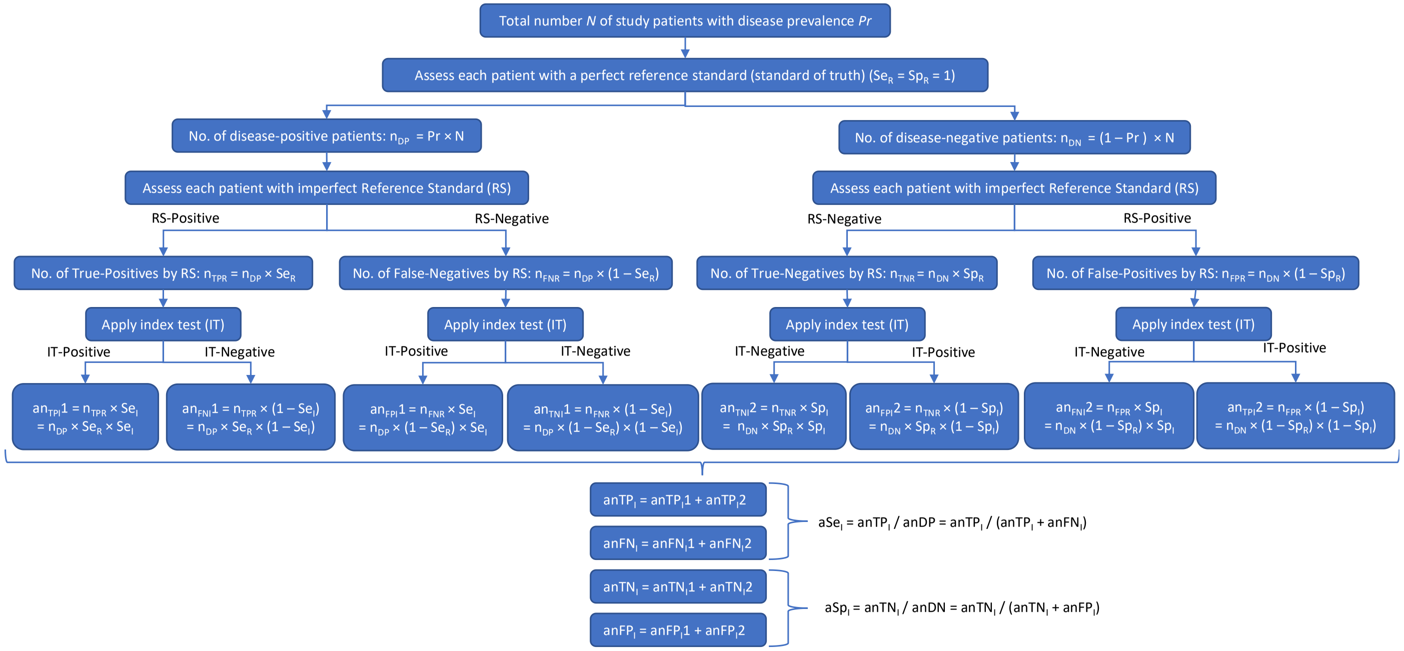

Figure S3. If the RS used is imperfect (Se_R and/or Sp_R <1), then subjects can have false-positive or false-negative results for the RS (FP_R or FN_R). Comparison of the results of an IT to the results of imperfect RS yields apparent results that may not be accurate. The apparent sensitivity and specificity of the IT (aSe_I and aSp_I) will depend on Se_R and Sp_R. For example, the apparent number of true positives (an_{TP}) will include all subjects who had a positive result for both the RS and the IT—even if they are disease-negative.

In Figure S3, variables are defined as follows:

- n_{DP} is the number of disease-positive patients,
- n_{DN} is the number of disease-negative patients
- n_{TPR} is the number of patients with true-positive RS results
- n_{FNR} is the number of patients with false-negative RS results
- n_{TNR} is the number of patients with true-negative RS results
- n_{FPR} is the number of patients with false-positive RS results
- Se_R is the sensitivity of the RS
- Sp_R is the specificity of the RS
- an_{DP} is the apparent number of disease-positive patients
- an_{DN} is the apparent number of disease-negative patients
- Se_I is the sensitivity of the IT
- Sp_I is the specificity of the IT
- $an_{TPI}1$ is the apparent number of true-positive IT results based on n_{TPR}
- $an_{TPI}2$ is the apparent number of true-positive IT results based on n_{FPR}
- $an_{FNI}1$ is the apparent number of false-negative IT results based on n_{TPR}
- $an_{FNI}2$ is the apparent number of false-negative IT results based on n_{FPR}
- $an_{FPI}1$ is the apparent number of false-positive IT results based on n_{FNR}
- $an_{FPI}2$ is the apparent number of false-positive IT results based on n_{TNR}
- $an_{TNI}1$ is the apparent number of true-negative IT results based on n_{FNR}
- $an_{TNI}2$ is the apparent number of true-negative IT results based on n_{TNR}
- an_{TPI} is the apparent total number of true-positive IT results
- an_{TNI} is the apparent total number of true-negative IT results

From Figure S3, Equations (S11) and (S12) (equivalent to Equations 13 and 14 in the manuscript) can be deduced:

$$an_{TPI} = n_{DP} \times Se_R \times Se_I + n_{DN} \times (1 - Sp_R) \times (1 - Sp_I) \tag{S11}$$

$$an_{TNI} = n_{DP} \times (1 - Se_R) \times (1 - Se_I) + n_{DN} \times Sp_R \times Sp_I \tag{S12}$$

If an_{TPI}, an_{TNI}, n_{DP}, n_{DN}, Se_R, and Sp_R are all known, then (S11) and (S12) constitute a system of equations with the 2 unknowns Se_I and Sp_I. Substituting single-letter variables and using the same online system-of-equations calculator referenced above allows solution. The variable substitutions were: $Se_I = x$, $Sp_I = y$, $an_{TPI} = a$, $n_{DP} = b$, $Se_R = c$, $n_{DN} = d$, $Sp_R = e$, and $an_{TNI} = f$. Rewriting with the new variables, (S11) becomes (S13):

$$a = b \times c \times x + d \times (1 - e) \times (1 - y) \tag{S13}$$

(S12) becomes (S14):

$$f = b \times (1 - c) \times (1 - x) + d \times e \times y \tag{S14}$$

This system of equations $a = b \times c \times x + d \times (1 - e) \times (1 - y), f = b \times (1 - c) \times (1 - x) + d \times e \times y$ was entered without the multiplication signs into an online calculator [1] to solve for x and y. The solutions were (S15) and (S16):

$$x = (ae - bce + bc + be - b + de^2 - de - ef + f) / b(c + e - 1) \tag{S15}$$

$$y = (-a \times c + a + b \times c^2 - b \times c - c \times d \times e + c \times d + c \times f + d \times e - d) / d \times (c + e - 1) \tag{S16}$$

Resubstituting the original variables gives (S17) and (S18), which are equivalent to Equations 15 and 16 in the manuscript:

$$Se_I = (an_{TPI} \times Sp_R - n_{DP} \times Se_R \times Sp_R + n_{DP} \times Se_R + n_{DP} \times Sp_R - n_{DP} + n_{DN} \times Sp_R^2 - n_{DN} \times Sp_R - Sp_R \times an_{TNI} + an_{TNI}) / (n_{DP} \times (Se_R + Sp_R - 1)) \tag{S17}$$

$$Sp_I = (-an_{TPI} \times Se_R + an_{TPI} + n_{DP} \times Se_R^2 - n_{DP} \times Se_R - Se_R \times n_{DN} \times Sp_R + Se_R \times n_{DN} + Se_R \times an_{TNI} + n_{DN} \times Sp_R - n_{DN}) / (n_{DN} \times (Se_R + Sp_R - 1)) \quad (S18)$$

As noted above, the term $Se_R + Sp_R - 1$ in the denominator is equal to Youden's J statistic.[2] Substituting J_R for this term may simplify further derivations in some circumstances (see below).

3. Derivations Using Proportions

The above formulas are in terms of patient counts. They can be converted to formulas that are based on proportions by dividing by N, the total number of patients in the study. For example, starting with Equation (S17), and dividing every term by N (equivalent to multiplying both the numerator and denominator by 1/N), one gets (S19):

$$Se_I = ((an_{TPI} \times Sp_R/N) - (n_{DP} \times Se_R \times Sp_R/N) + (n_{DP} \times J_R/N) + (n_{DN} \times Sp_R^2/N) - (n_{DN} \times Sp_R/N) - (Sp_R \times an_{TNI}/N) + (an_{TNI}/N)) / (n_{DP} \times J_R/N) \quad (S19)$$

Since n_{DP} / N = Pr (the prevalence of the index disease), and since n_{DN} / N = 1 − Pr, one can rewrite (S19) as (S20) (shown as Equation 22 in the manuscript):

$$Se_I = (pa_{TPI} \times Sp_R - Pr \times Se_R \times Sp_R + Pr \times J_R + (1 - Pr) \times Sp_R^2 - (1 - Pr) \times Sp_R - Sp_R \times pa_{TNI} + pa_{TNI}) / Pr \times J_R \quad (S20)$$

In (S20), pa_{TPI} is the proportion of patients with an apparently true-positive IT result, Pr is the prevalence of the index disease, J_R is Youden's Index for the RS, pa_{TNI} is the proportion of patients with an apparently true-negative IT result, Sp_R is the specificity of the RS, and Se_R is the sensitivity of the RS.

Similarly, one can divide every term in (S18) by N to get (S21) (shown as Equation 23 in the manuscript):

$$Sp_I = (pa_{TPI} - pa_{TPI} \times Se_R + Pr \times Se_R^2 - Pr \times Se_R - Se_R \times (1 - Pr) \times Sp_R + (1 - Pr) \times J_R + Se_R \times pa_{TNI}) / ((1 - Pr) \times J_R) \quad (S21)$$

In (S21), the variables have the same meaning as in (S20).

4. Investigational Test Apparent Sensitivity and Specificity

Although not part of the study objectives, formulas showing the relationship between n_{DP}, n_{DN}, Se_R, Sp_R, Se_I, and Sp_I and the apparent sensitivity and specificity of the investigational test (aSe_I and aSp_I) were deduced from Figure S3, as shown below. This was done for completeness and to allow comparison of my formulas with those of prior authors to determine if the formulas are equivalent.

4.1.1 Investigational Test Apparent Sensitivity

Using the above relationships, one can derive formulas relating the apparent values of the IT's Se and Sp (aSe_I and aSp_I), which are observed in a clinical study that uses an imperfect RS, to the true values of the IT's Se and Sp (Se_I and Sp_I), and the values of n_{TPR}, n_{TNR}, n_{FPR}, and n_{FNR} (or, equivalently, Se_R, Sp_R, n_{DP}, and n_{DN}).

Since sensitivity is the number of patients with true-positive IT results (n_{TPI}) divided by the number of disease-positive patients (n_{DP}), aSe_I equals the apparent number of true-positive IT results (an_{TPI}) divided by the apparent number of disease-positive patients (an_{DP}):

$$aSe_I = an_{TPI} / an_{DP} \quad (S22)$$

Above, I derived formulas for an_{TPI} in terms of n_{DP}, n_{DN}, Se_R, Sp_R, Se_I, and Sp_I (S11), and for an_{DP} in terms of n_{DP}, n_{DN}, Se_R, and Sp_R (S3). Dividing (S11) by (S3) gives a formula for aSe_I in terms of n_{DP}, n_{DN}, Se_R, Sp_R, Se_I, and Sp_I:

$$aSe_I = an_{TPI} / an_{DP} = (n_{DP} \times Se_R \times Se_I + n_{DN} \times (1 - Sp_R) \times (1 - Sp_I)) / (n_{DP} \times Se_R + n_{DN} \times (1 - Sp_R)) \quad \text{(S23)}$$

As a simple validity check, if $Se_R = Sp_R = 1$, then (S23) should simplify to $aSe_I = Se_I$:

$$aSe_I = (n_{DP} \times 1 \times Se_I + n_{DN} \times (1 - 1) \times (1 - Sp_I)) / (n_{DP} \times 1 + n_{DN} \times (1 - 1)) \quad \text{(S24)}$$

The n_{DN} terms in (S24) become 0, the n_{DP} terms in the numerator and denominator cancel, and the equation reduces to:

$$aSe_I = Se_I \quad \text{(S25)}$$

As expected, this indicates that when the reference standard is perfect ($Se_R = Sp_R = 1$), then there is no patient misclassification, and the apparent sensitivity of the investigational test (aSe_I) that is observed in a clinical trial is the true sensitivity (Se_I).

4.1.2 Investigational Test Apparent Specificity

Since specificity is the number of true-negative IT results (n_{TNI}) divided by the number of disease-negative patients (n_{DN}), aSp_I equals the apparent number of true-negative IT results (an_{TNI}) divided by the apparent number of disease-negative patients (an_{DN}):

$$aSp_I = an_{TNI} / an_{DN} \quad \text{(S26)}$$

Above, I derived formulas for an_{TNI} in terms of n_{DP}, n_{DN}, Se_R, Sp_R, Se_I, and Sp_I (S12), and for an_{DN} in terms of n_{DP}, n_{DN}, Se_R, and Sp_R (S4). Dividing (S12) by (S4) gives a formula for aSp_I in terms of n_{DP}, n_{DN}, Se_R, Sp_R, Se_I, and Sp_I:

$$aSp_I = an_{TNI} / an_{DN} = (n_{DP} \times (1 - Se_R) \times (1 - Se_I) + n_{DN} \times Sp_R \times Sp_I) / (n_{DP} \times (1 - Se_R) + n_{DN} \times Sp_R) \quad \text{(S27)}$$

As a simple check, if $Se_R = Sp_R = 1$, then (S27) should simplify to $aSp_I = Sp_I$:

$$aSp_I = (n_{DP} \times (1 - 1) \times (1 - Se_I) + n_{DN} \times 1 \times Sp_I) / (n_{DP} \times (1 - 1) + n_{DN} \times 1) \quad \text{(S28)}$$

The n_{DP} terms become 0, the n_{DN} terms in the numerator and denominator cancel, and the equation reduces to:

$$aSp_I = Sp_I \quad \text{(S29)}$$

As expected, this indicates that when the reference standard is perfect ($Se_R = Sp_R = 1$), then the apparent specificity of the investigational test (aSp_I) that is observed in a clinical trial is the true specificity (Sp_I).

Using Equations (S23) and (S27), the impact of an imperfect RS on the apparent Se and Sp of an investigational test (aSe_I and aSp_I, respectively) can be estimated.

4.1.3 Impact on the Apparent Sensitivity of an Investigational Test

Equation (S23) gives the apparent sensitivity of an investigational test that would result from use of an imperfect RS:

$$aSe_I = an_{TPI} / an_{DP} = (n_{DP} \times Se_R \times Se_I + n_{DN} \times (1 - Sp_R) \times (1 - Sp_I)) / (n_{DP} \times Se_R + n_{DN} \times (1 - Sp_R)) \quad \text{(S23)}$$

To determine that impact that an imperfect RS may have on aSe_I, let's assume that the investigational test being validated is perfect, i.e., $Se_I = Sp_I = 1$. Let's also assume that the number of patients with the index disease is the same as the number of patients without the disease (i.e., the disease prevalence in the study is 0.5). Then, from Equation (S23):

$$aSe_I = (n_{DP} \times Se_R) / (n_{DP} \times Se_R + n_{DN} \times (1 - Sp_R)) \quad \text{(S30)}$$

$$aSe_I = (Se_R) / (Se_R + (1 - Sp_R)) \quad \text{(S31)}$$

Se_R appears in both the numerator and the denominator. The denominator also contains the term $1 - Sp_R$. If Sp_R is 1, then the $1 - Sp_R$ term will be 0, and then aSe_I will be Se_R/Se_R, or 1, which equals the assumed value of Se_I. However, if Sp_R is less than 1, then

the 1 – Sp_R term will be greater than 0, resulting in aSe_I values that are less than 1. Thus, an imperfect value of Sp_R will decrease aSe_I from the true value (Se_I). In summary, the apparent sensitivity of the investigational test (aSe_I) is controlled mainly by Sp_R, the specificity of the RS. More specifically, it is controlled by 1 – Sp_R, which equals the false positive rate (FPR) of the RS.

Figure S4 shows a plot of the apparent sensitivity of the investigational test (aSe_I) versus the specificity of the reference standard (Sp_R), for two values of the sensitivity of the RS (Se_R), 1.0 and 0.5. This figure shows that Sp_R has a much greater effect on aSe_I than does Se_R. The figure also shows that an RS with Sp_R less than 1 can result in a substantial reduction of the apparent Se of an investigational test (aSe_I), even if the investigational test is perfect ($Se_I = Sp_I = 1$).

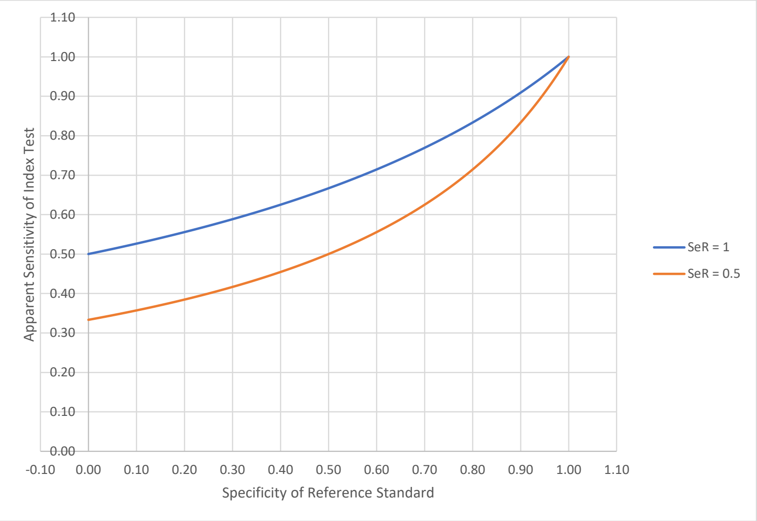

Figure S4. Low specificity of a reference standard would reduce the apparent sensitivity of a perfect index test.

Equation (S27) gives the apparent specificity of an investigational test that would result from use of an imperfect RS:

$$aSp_I = an_{TNI} / an_{DN} = (n_{DP} \times (1 - Se_R) \times (1 - Se_I) + n_{DN} \times Sp_R \times Sp_I) / (n_{DP} \times (1 - Se_R) + n_{DN} \times Sp_R) \quad (S27)$$

To determine that impact that an imperfect RS may have on aSp_I, let's again assume that the investigational test being validated is perfect, i.e., $Se_I = Sp_I = 1$. Let's also assume (again) that the number of patients with disease is the same as the number of patients without the disease (i.e., the disease prevalence in the study is 0.5). Then, from Equation (S27):

$$aSp_I = ((1 - Se_R) \times (1 - 1) + Sp_R \times 1) / ((1 - Se_R) + Sp_R) \quad (S32)$$

$$aSp_I = (Sp_R) / (Sp_R + (1 - Se_R)) \quad (S33)$$

This shows that the apparent specificity of the investigational test (aSe_I) is controlled mainly by Se_R, the sensitivity of the RS. More specifically, it is controlled by 1 – Se_R, which equals the false negative rate (FNR) of the RS. Figure S5 shows a plot of the apparent specificity of the investigational test (aSp_I) versus the sensitivity of the reference standard (Se_R), for two values of the specificity of the RS (Sp_R), 1.0 and 0.5. This figure shows that Se_R has a much greater effect on aSp_I than does Sp_R. The figure also shows that a RS with Se_R less than 1 can result in a substantial reduction of the apparent Sp of an investigational test (aSp_I), even if the investigational test is perfect ($Se_I = Sp_I = 1$).

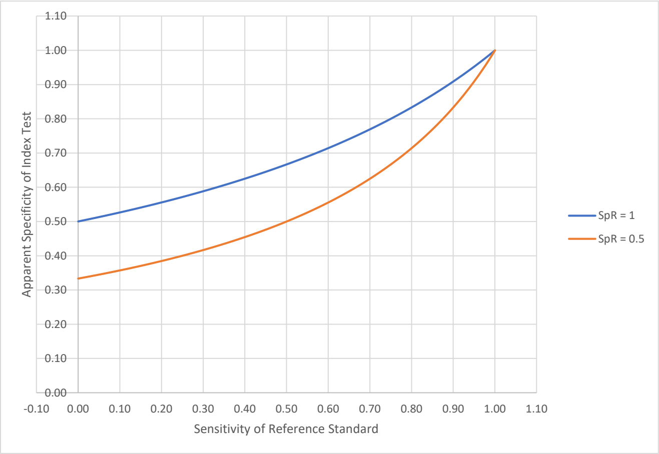

Figure S5. Low sensitivity of a reference standard would reduce the apparent specificity of a perfect index test.

The above simulations help illustrate the need for a method to correct for the effects of an imperfect RS.

5. Comparisons to Prior Work

Two publications [3,4] reported derivation of equations for calculating Se_I and Sp_I (or aSe_I and aSp_I) when an imperfect RS is used. These publications took different approaches than I did and reported different formulas. Because they gave insufficient detail to allow confirmation of their work, I attempted to show that their formulas were equivalent to mine.

5.1 Gart and Buck 1996

5.1.1 Investigational Test Sensitivity

Gart and Buck [3] discussed the use of screening and reference tests for estimating disease prevalence in epidemiologic studies. They derived formulas for what they termed co-positivity and co-negativity (which I determined to be equivalent to aSe_I and aSp_I), and solved these for Se_I and Sp_I if Pr, Se_R and Sp_R, aSe_I and aSp_I are known. I was able to show that my formulas for Se_I and Sp_I (after transformation into proportion-based variables) were equivalent to theirs, as follows. They referred to the true sensitivity of a screening test as S_1'. Their formula for S_1' is:

$$S1' = (S2 \times Prr \times C1 + (1 - S2) \times (1 - Prr) \times C2 - (1 - S2) \times (S2 - Pr \times J)) / J \times Pr \qquad (S34)$$

In (S34), S_1' = sensitivity of the screening test (which I denote as Se_I), S_2 = specificity of the reference standard (RS) (which I denote as Sp_R), Prr = apparent prevalence based on the RS (which I denote as aPr), C_1 = apparent Se of the screening test (which I denote as aSe_I), C_2 = apparent Sp of the screening test (which I denote as aSp_I), Pr = true prevalence, J = Youden's Index for RS (which I denote as J_R; $J_R = Se_R + Sp_R - 1$). Substituting my variables for theirs:

$$Se_I \text{ (Gart \& Buck)} = (Sp_R \times aPr \times aSe_I + (1 - Sp_R) \times (1 - aPr) \times aSp_I - (1 - Sp_R) \times (Sp_R - Pr \times J_R)) / J_R \times Pr \qquad (S35)$$

Since Gart and Buck's formula is based on proportions and Youden's Index, I needed to compare it to my formula that is similarly based. Above, I converted (S17) for Se_I in terms of patient counts to the proportion-based version using Youden's Index (S20) by dividing each term in (S17) by N (the total number of patients), and by substituting J_R for $Se_R + Sp_R - 1$.

In (S35), the term $aPr \times aSe_I$ is equal to my term pa_{TPI} in (S20), and their term $(1 - aPr) \times (aSp_I)$ is equal to my term pa_{TNI}. Making these substitutions:

$$Se_I \text{ (Gart \& Buck)} = (Sp_R \times pa_{TPI} + (1 - Sp_R) \times pa_{TNI} - (1 - Sp_R) \times (Sp_R - Pr \times J_R)) / (J_R \times Pr) \qquad (S36)$$

By comparing (S36) to (S20), one can see that the denominators are equal, so if it can be shown that the numerators are equal then the 2 equations will be equal. Substituting the following simpler variables into each equation allows use of an online expansion calculator:

$$J_R = a,\ pa_{TPI} = b,\ pa_{TNI} = c,\ Pr = d,\ Se_R = e,\ Sp_R = f$$

The Gart & Buck numerator thus becomes:

$$\text{Gart \& Buck numerator} = f \times b + (1-f) \times c - (1-f) \times (f - da) \tag{S37}$$

After expansion [5], this becomes:

$$\text{Gart \& Buck numerator} = f \times b + c - f \times c - f + d \times a + f^2 - f \times d \times a \tag{S38}$$

The Sherwin numerator in (S27) becomes:

$$b \times f - d \times e \times f + d \times a + (1-d) \times f^2 - (1-d) \times f - f \times c + c \tag{S39}$$

After expansion [5] this becomes:

$$b \times f - d \times e \times f + d \times a + f^2 - f^2 \times d - f + f \times d - f \times c + c \tag{S40}$$

Setting the 2 numerators equal:

$$f \times b + c - f \times c - f + d \times a + f^2 - f \times d \times a = b \times f - d \times e \times f + d \times a + f^2 - f^2 \times d - f + f \times d - f \times c + c \tag{S41}$$

Eliminating terms that appear on both sides gives:

$$\cancel{f} \times \cancel{b} + \cancel{c} - \cancel{f} \times \cancel{c} - \cancel{f} + \cancel{d} \times \cancel{a} + \cancel{f^2} - f \times d \times a = \cancel{b} \times \cancel{f} - d \times e \times f + \cancel{d} \times \cancel{a} + \cancel{f^2} - f^2 \times d - \cancel{f} + f \times d - \cancel{f} \times \cancel{c} + \cancel{c} \tag{S42}$$

$$-f \times d \times a = -d \times e \times f - f^2 \times d + f \times d \tag{S43}$$

Dividing both sides by $d \times f$ gives:

$$-a = -e - f + 1 \tag{S44}$$

Multiplying both sides by −1 gives:

$$a = e + f - 1 \tag{S45}$$

Resubstituting the original variables gives:

$$J_R = Se_R + Sp_R - 1 \tag{S46}$$

(S46) is simply the definition of J_R, so the 2 numerators and thus the 2 equations are equivalent.

5.1.2 Investigational Test Specificity

Gart and Buck [3] referred to the true specificity of a screening test as S_2'. Their formula for S_2' is:

$$S_2' = (S_1 \times (1 - Prr) \times C_2 + (1 - S_1) \times Prr \times C_1 - (1 - S_1) \times (1 - S_2 + Pr \times J)) / (J \times (1 - Pr)) \tag{S47}$$

In (S47), S_2' = specificity of the screening test (Sp_I); S_1 = sensitivity of the reference standard (RS) (Se_R); Prr = apparent prevalence based on the RS (aPr); C_2 = apparent specificity of the screening test (aSp_I); C_1 = apparent Se of the screening test (aSe_I); S_2 = specificity of the RS (Sp_R); Pr = true prevalence; J = Youden's Index for reference standard (RS) ($J_R = Se_R + Sp_R - 1$).

Substituting my variables for theirs:

$$Sp_I\ (\text{Gart \& Buck}) = (Se_R \times (1 - aPr) \times aSp_I + (1 - Se_R) \times aPr \times aSe_I - (1 - Se_R) \times (1 - Sp_R + Pr \times J_R)) / (J_R \times (1 - Pr)) \tag{S48}$$

Since Gart and Buck's formula is based on proportions and Youden's Index, I needed to compare it to my formula that is similarly based. Above, I converted (S18) (based on patient counts) to the proportion-based (S21) using Youden's Index by dividing each term by N (the total number of patients) and by substituting J_R for $Se_R + Sp_R - 1$.

In (S48), the term *(1 − aPr) × aSp*$_I$ is equal to my term pa_{TNI} in (S21), and their term $aPr \times aSe_I$ is equal to my term pa_{TPI}. Making these substitutions:

$$Sp_I \text{ (Gart \& Buck)} = (Se_R \times pa_{TNI} + (1 - Se_R) \times pa_{TPI} - (1 - Se_R) \times (1 - Sp_R + Pr \times J_R)) / (J_R \times (1 - Pr)) \quad \text{(S49)}$$

By comparing (S49) to (S21), one can see that the denominators are equal, so if it can be shown that the numerators are equal, then the 2 equations will be equal. Substituting the same simpler variables as used immediately above into each formula to allow use of an online expansion calculator:

$$J_R = a,\ pa_{TPI} = b,\ pa_{TNI} = c,\ Pr = d,\ Se_R = e,\ Sp_R = f$$

After substitutions, the Gart & Buck numerator becomes:

$$Sp_I \text{ (Gart \& Buck)} = e \times c + (1 - e) \times b - (1 - e) \times (1 - f + d \times a) \quad \text{(S50)}$$

After expansion, this becomes:

$$\text{Gart \& Buck numerator} = e \times c + b - e \times b - 1 + f - d \times a + e - e \times f + e \times d \times a \quad \text{(S51)}$$

After substitutions, the Sherwin numerator in (S21) becomes:

$$\text{Sherwin numerator} = b - b \times e + d \times e^2 - d \times e - e \times (1 - d) \times f + (1 - d) \times a + e \times c \quad \text{(S52)}$$

After expansion [5] this becomes:

$$\text{Sherwin numerator} = b - b \times e + d \times e^2 - d \times e - e \times f + e \times d \times f + a - d \times a + e \times c \quad \text{(S53)}$$

Setting the 2 numerators equal:

$$e \times c + b - e \times b - 1 + f - d \times a + e - e \times f + e \times d \times a = b - b \times e + d \times e^2 - d \times e - e \times f + e \times d \times f + a - d \times a + e \times c \quad \text{(S54)}$$

Eliminating terms that appear on both sides gives:

$$\cancel{e} \times \cancel{c} + \cancel{b} - \cancel{e} \times \cancel{b} - 1 + f - \cancel{d} \times \cancel{a} + e - \cancel{e} \times \cancel{f} + e \times d \times a = \cancel{b} - \cancel{b} \times \cancel{e} + d \times e^2 - d \times e - \cancel{e} \times \cancel{f+} e \times d \times f + a - \cancel{d} \times \cancel{a} + \cancel{e} \times \cancel{c} \quad \text{(S55)}$$

$$-1 + f + e + e \times d \times a = d \times e^2 - d \times e + e \times d \times f + a \quad \text{(S56)}$$

Since $a = J_R$, $e = Se_R$, $f = Sp_R$, and $J_R = Se_R + Sp_R - 1$, then on the left side one can substitute a for $e + f - 1$:

$$a + e \times d \times a = d \times e^2 - d \times e + e \times d \times f + a \quad \text{(S57)}$$

$$e \times d \times a = d \times e^2 - d \times e + e \times d \times f \quad \text{(S58)}$$

Dividing both sides by $e \times d$:

$$a = e - 1 + f \quad \text{(S59)}$$

(S59) is equivalent to the definition of J_R, so the 2 numerators and thus the 2 formulas are equivalent.

5.2 *Staquet et al. 1981*

Staquet et al [4] derived formulas to correct for misclassification by a reference standard. They denoted the true Se of the new (investigational) test (which I call Se_I) as S_N, the true Sp of the investigational test (which I call Sp_I) as SP_N, the Se of the reference standard (which I call Se_R) as S_R, and the Sp of the reference standard (which I call Sp_R)

as SP_R. They reported the following formulas, which use counts rather than proportions of patients:

$$S_N = Se_I = ((a+c) \times SP_R - c) / (N \times (SP_R - 1) + a + b) \quad (S60)$$

$$SP_N = Sp_I = ((b+d) \times S_R - b) / ((N \times S_R) - (a+b)) \quad (S61)$$

From Table 2 in their article, it can be deduced that $a = an_{TPI}$, $b = an_{FNI}$, $c = an_{FPI}$, and $d = an_{TNI}$; also, their variable S_R is the same as my variable Se_R and their variable SP_R is the same as my variable Sp_R. Substituting my variables for S_R and SP_R into (S60) and (S61):

$$Se_I \text{ (Staquet)} = ((a+c) \times Sp_R - c) / (N \times (Sp_R - 1) + a + b) \quad (S62)$$

$$Sp_I \text{ (Staquet)} = ((b+d) \times Se_R - b) / (N \times Se_R - (a+b)) \quad (S63)$$

All of the variables should be converted to single-letter variables (to allow use of an online equation expander). Letting $Se_R = f$, $Sp_R = g$, and N = n:

$$Se_I \text{ (Staquet)} = ((a+c) \times g - c)/(n \times (g-1) + (a+b)) \quad (S64)$$

$$Sp_I \text{ (Staquet)} = ((b+d) \times f - b) / (n \times f - (a+b)) \quad (S65)$$

Expanding using an online calculator [5]:

$$Se_I \text{ (Staquet)} = (a \times g + c \times g - c) / (n \times g - n + \mathrm{a} + b) \quad (S66)$$

$$Sp_I \text{ (Staquet)} = (b \times f + d \times f - b) / (n \times f - a - b) \quad (S67)$$

Substituting my variables for theirs in (S66) and (S67) yields (S68) and (S69):

$$Se_I \text{ (Staquet)} = (an_{TPI} \times Sp_R + an_{FPI} \times Sp_R - an_{FPI}) / (N \times Sp_R - N + an_{TPI} + an_{FNI}) \quad (S68)$$

$$Sp_I \text{ (Staquet)} = (an_{FNI} \times Se_R + an_{TNI} \times Se_R - an_{FNI}) / (N \times Se_R - an_{TPI} - an_{FNI}) \quad (S69)$$

Focusing first on (S68), substituting $an_{DP} + an_{DN}$ for N and an_{DP} for $an_{TPI} + an_{FNI}$:

$$Se_I \text{ (Staquet)} = (an_{TPI} \times Sp_R + an_{FPI} \times Sp_R - an_{FPI}) / ((an_{DP} + an_{DN}) \times Sp_R - (an_{DP} + an_{DN}) + an_{DP}) \quad (S70)$$

Canceling terms:

$$Se_I \text{ (Staquet)} = (an_{TPI} \times Sp_R + an_{FPI} \times Sp_R - an_{FPI}) / (an_{DP} \times Sp_R + an_{DN} \times Sp_R - \cancel{an_{DP}} - an_{DN} + \cancel{an_{DP}}) \quad (S71)$$

Gives:

$$Se_I \text{ (Staquet)} = (an_{TPI} \times Sp_R + an_{FPI} \times Sp_R - an_{FPI}) / (an_{DP} \times Sp_R + an_{DN} \times Sp_R - an_{DN}) \quad (S72)$$

Now focusing on (S69), substituting $an_{DP} + an_{DN}$ for N and an_{DP} for $an_{TPI} + an_{FNI}$:

$$Sp_I \text{ (Staquet)} = (an_{FNI} \times Se_R + an_{TNI} \times Se_R - an_{FNI}) / ((an_{DP} + an_{DN}) \times Se_R - an_{DP}) \quad (S73)$$

Expanding terms gives:

$$Sp_I \text{ (Staquet)} = (an_{FNI} \times Se_R + an_{TNI} \times Se_R - an_{FNI}) / (an_{DP} \times Se_R + an_{DN} \times Se_R - an_{DP}) \quad (S74)$$

To compare (S72) and (S74) to my formulas for Se_I and Sp_I, I needed to re-derive my formulas in terms of an_{DP} and an_{DN}. Above, I derived formulas for aSe_I (S23) and aSp_I (S27). Since one cannot directly determine n_{DP} and n_{DN} in a clinical trial, one must substitute the expressions for n_{DP} and n_{DN} in terms of an_{DP} and an_{DN} (see (S9) and (S10)) into (S23). Making those substitutions as well as subsituting J_R for $Se_R + Sp_R - 1$ gives (S75):

$$aSe_I = (((an_{DP} \times Sp_R + Sp_R \times an_{DN} - an_{DN}) / J_R) \times Se_R \times Se_I + ((an_{DP} \times Se_R - an_{DP} + Se_R \times an_{DN}) / J_R) \times (1 - Sp_R) \times (1 - Sp_I)) / (((an_{DP} \times Sp_R + Sp_R \times an_{DN} - an_{DN}) / J_R) \times Se_R + ((an_{DP} \times Se_R - an_{DP} + Se_R \times an_{DN}) / J_R) \times (1 - Sp_R)) \quad (S75)$$

To expand the formula, one can substitute simpler variables and then expand by using an online calculator [5]: $an_{DP} = a$, $an_{DN} = b$, $Se_R = c$, $Sp_R = d$, $J_R = e$, $Se_I = x$, $Sp_I = y$.

$$aSe_I = (((a \times d + d \times b - b) / e) \times c \times x + ((a \times c - a + c \times b) / e) \times (1 - d) \times (1 - y)) / (((a \times d + d \times b - b) / e) \times c + ((a \times c - a + c \times b) / e) \times (1 - d)) \quad \text{(S76)}$$

Expanding:

$$aSe_I = (a \times d \times c \times x + d \times b \times c \times x - b \times c \times x + a \times c - a \times c \times y - a \times d \times c + a \times d \times c \times y - a + a \times y + a \times d - a \times d \times y + b \times c - b \times c \times y - d \times b \times c + d \times b \times c \times y) / (a \times c - a + a \times d) \quad \text{(S77)}$$

Substituting back the more meaningful variables:

$an_{DP} = a$, $an_{DN} = b$, $Se_R = c$, $Sp_R = d$, $Se_I = x$, $Sp_I = y$

$$aSe_I = (an_{DP} \times Sp_R \times Se_R \times Se_I + Sp_R \times an_{DN} \times Se_R \times Se_I - an_{DN} \times Se_R \times Se_I + an_{DP} \times Se_R - an_{DP} \times Se_R \times Sp_I - an_{DP} \times Sp_R \times Se_R + an_{DP} \times Sp_R \times Se_R \times Sp_I - an_{DP} + an_{DP} \times Sp_I + an_{DP} \times Sp_R - an_{DP} \times Sp_R \times Sp_I + an_{DN} \times Se_R - an_{DN} \times Se_R \times Sp_I - Sp_R \times an_{DN} \times Se_R + Sp_R \times an_{DN} \times Se_R \times Sp_I) / (an_{DP} \times Se_R - an_{DP} + an_{DP} \times Sp_R) \quad \text{(S78)}$$

For the specificity of the investigational test, one can start with (S27). Since one can't directly determine the values of n_{DP} and n_{DN} in a clinical trial of an investigational test, one must substitute the expressions for n_{DP} and n_{DN} ((S9) and (S10)) into (S27). Making those substitutions as well as subsituting J_R for $Se_R + Sp_R - 1$ gives (S79):

$$aSp_I = (((an_{DP} \times Sp_R + Sp_R \times an_{DN} - an_{DN})/J_R) \times (1 - Se_R) \times (1 - Se_I) + ((an_{DP} \times Se_R - an_{DP} + Se_R \times an_{DN})/J_R) \times Sp_R \times Sp_I) / (((an_{DP} \times Sp_R + Sp_R \times an_{DN} - an_{DN})/J_R) \times (1 - Se_R) + ((an_{DP} \times Se_R - an_{DP} + Se_R \times an_{DN}) / J_R) \times Sp_R) \quad \text{(S79)}$$

To expand the right side of the formula, one can substitute simpler variables: $an_{DP} = a$, $an_{DN} = b$, $Se_R = c$, $Sp_R = d$, $J_R = e$, $Se_I = x$, $Sp_I = y$.

$$aSp_I = (((a \times d + d \times b - b) / e) \times (1 - c) \times (1 - x) + ((a \times c - a + c \times b) / e) \times d \times y) / (((a \times d + d \times b - b) / e) \times (1 - c) + ((a \times c - a + c \times b) / e) \times d) \quad \text{(S80)}$$

Expanding the right side by using an online calculator [5]:

$$aSp_I = (a \times d - a \times d \times x - a \times d \times c + a \times d \times c \times x + d \times b - d \times b \times x - d \times b \times c + d \times b \times c \times x - b + b \times x + b \times c - b \times c \times x + a \times d \times c \times y - a \times d \times y + d \times b \times c \times y) / (d \times b - b + b \times c) \quad \text{(S81)}$$

Substituting back the more meaningful variables:

$an_{DP} = a$ $an_{DN} = b$ $Se_R = c$ $Sp_R = d$ $Se_I = x$ $Sp_I = y$

$$aSp_I = (an_{DP} \times Sp_R - an_{DP} \times Sp_R \times Se_I - an_{DP} \times Sp_R \times Se_R + an_{DP} \times Sp_R \times Se_R \times Se_I + Sp_R \times an_{DN} - Sp_R \times an_{DN} \times Se_I - Sp_R \times an_{DN} \times Se_R + Sp_R \times an_{DN} \times Se_R \times Se_I - an_{DN} + an_{DN} \times Se_I + an_{DN} \times Se_R - an_{DN} \times Se_R \times Se_I + an_{DP} \times Sp_R \times Se_R \times Sp_I - an_{DP} \times Sp_R \times Sp_I + Sp_R \times an_{DN} \times Se_R \times Sp_I) / (Sp_R \times an_{DN} - an_{DN} + an_{DN} \times Se_R) \quad \text{(S82)}$$

The above formulas for aSe_I and aSp_I ((S78) and (S82), respectively) constitute a system of 2 equations with the 2 unknowns Se_I and Sp_I. One can use an online calculator to solve them, but first one must simplify them by substituting single-letter variables. However, I already did this to simplify the formulas for aSe_I and aSp_I (see (S78) and (S81), respectively), with the following variable definitions: $an_{DP} = a$, $an_{DN} = b$, $Se_R = c$, $Sp_R = d$, $Se_I = x$, $Sp_I = y$. One just needs to select simpler variables for aSe_I and aSp_I, which I will denote as f and g respectively:

$$aSe_I = f = (a \times d \times c \times x + d \times b \times c \times x - b \times c \times x + a \times c - a \times c \times y - a \times d \times c + a \times d \times c \times y - a + a \times y + a \times d - a \times d \times y + b \times c - b \times c \times y - d \times b \times c + d \times b \times c \times y) / (a \times c - a + a \times d) \tag{S83}$$

$$aSp_I = g = (a \times d - a \times d \times x - a \times d \times c + a \times d \times c \times x + d \times b - d \times b \times x - d \times b \times c + d \times b \times c \times x - b + b \times x + b \times c - b \times c \times x + a \times d \times c \times y - a \times d \times y + d \times b \times c \times y) / (d \times b - b + b \times c) \tag{S84}$$

According to an online equation solver [1] the solutions are:

$$x = (a \times d \times f - b \times d \times g + b \times d + b \times g - b) / (a \times d + b \times d - b) \tag{S85}$$

And...

$$y = (-a \times c \times f + a \times c + a \times f - a + b \times c \times g) / (a \times c - a + b \times c) \tag{S86}$$

Substituting back the more meaningful variables:

$$an_{DP} = a,\ an_{DN} = b,\ Se_R = c,\ Sp_R = d,\ Se_I = x,\ Sp_I = y,\ aSe_I = f,\ aSp_I = g$$

$$Se_I = (an_{DP} \times Sp_R \times aSe_I - an_{DN} \times Sp_R \times aSp_I + an_{DN} \times Sp_R + an_{DN} \times aSp_I - an_{DN}) / (an_{DP} \times Sp_R + an_{DN} \times Sp_R - an_{DN}) \tag{S87}$$

And...

$$Sp_I = (an_{DN} \times Se_R \times aSp_I - an_{DP} \times Se_R \times aSe_I + an_{DP} \times Se_R + an_{DP} \times aSe_I - an_{DP}) / (an_{DP} \times Se_R + an_{DN} \times Se_R - an_{DP}) \tag{S88}$$

Since $aSe_I = an_{TPI} / an_{DP}$ and $aSp_I = an_{TNI} / an_{DN}$, one can substitute these into (S87) and (S88) and cancel some terms. Starting with (S87):

$$Se_I = (\cancel{an_{DP} \times} Sp_R \times an_{TPI}/\cancel{an_{DP}} - \cancel{an_{DN} \times} Sp_R \times an_{TNI}/\cancel{an_{DN}} + an_{DN} \times Sp_R + \cancel{an_{DN} \times} an_{TNI} /\cancel{an_{DN}} - an_{DN}) / (an_{DP} \times Sp_R + an_{DN} \times Sp_R - an_{DN}) \tag{S89}$$

$$Se_I = (Sp_R \times an_{TPI} - Sp_R \times an_{TNI} + an_{DN} \times Sp_R + an_{TNI} - an_{DN}) / (an_{DP} \times Sp_R + an_{DN} \times Sp_R - an_{DN}) \tag{S90}$$

Similarly, after substitution into (S88) and cancelling:

$$Sp_I = (\cancel{an_{DN} \times} Se_R \times an_{TNI}/\cancel{an_{DN}} - \cancel{an_{DP} \times} Se_R \times an_{TPI} /\cancel{an_{DP}} + an_{DP} \times Se_R + \cancel{an_{DP} \times} an_{TPI}/\cancel{an_{DP}} - an_{DP}) / (an_{DP} \times Se_R + an_{DN} \times Se_R - an_{DP}) \tag{S91}$$

$$Sp_I = (Se_R \times an_{TNI} - Se_R \times an_{TPI} + an_{DP} \times Se_R + an_{TPI} - an_{DP}) / (an_{DP} \times Se_R + an_{DN} \times Se_R - an_{DP}) \tag{S92}$$

Now one can compare my formulas to Staquet's.[4]

5.2.1 Investigational Test Sensitivity

With respect to **sensitivity**, comparing (S71) to (S90), the denominators are equal. Therefore, if the numerators are equal, the formulas will be. Setting the numerators equal:

$$an_{TPI} \times Sp_R + an_{FPI} \times Sp_R - an_{FPI} = Sp_R \times an_{TPI} - Sp_R \times an_{TNI} + an_{DN} \times Sp_R + an_{TNI} - an_{DN} \tag{S93}$$

Eliminating terms that appear on both sides:

$$\cancel{an_{TPI} \times Sp_R +} an_{FPI} \times Sp_R - an_{FPI} = \cancel{Sp_R \times an_{TPI}} - Sp_R \times an_{TNI} + an_{DN} \times Sp_R + an_{TNI} - an_{DN} \tag{S94}$$

$$an_{FPI} \times Sp_R - an_{FPI} = -Sp_R \times an_{TNI} + an_{DN} \times Sp_R + an_{TNI} - an_{DN} \tag{S95}$$

Rearranging terms on the right side:

$$an_{FPI} \times Sp_R - an_{FPI} = Sp_R \times (-an_{TNI} + an_{DN}) + (an_{TNI} - an_{DN}) \quad (S96)$$

Since $an_{DN} - an_{TNI} = an_{FPI}$ then one can write (S96) as:

$$an_{FPI} \times Sp_R - an_{FPI} = an_{FPI} \times Sp_R - an_{FPI} \quad (S97)$$

Which shows equality of the numerators. Therefore, the formulas are equal.

5.2.2 Investigational Test Specificity

With respect to **specificity**, comparison of (S73) and (S92) shows equality of the denominators. Setting the numerators equal to each other:

$$an_{FNI} \times Se_R + an_{TNI} \times Se_R - an_{FNI} = Se_R \times an_{TNI} - Se_R \times an_{TPI} + an_{DP} \times Se_R + an_{TPI} - an_{DP} \quad (S98)$$

Eliminating terms that appear on both sides:

$$an_{FNI} \times Se_R + \cancel{an_{TNI} \times Se_R} - an_{FNI} = \cancel{Se_R \times an_{TNI}} - Se_R \times an_{TPI} + an_{DP} \times Se_R + an_{TPI} - an_{DP} \quad (S99)$$

$$an_{FNI} \times Se_R - an_{FNI} = -Se_R \times an_{TPI} + an_{DP} \times Se_R + an_{TPI} - an_{DP} \quad (S100)$$

Rearranging terms on the right side:

$$an_{FNI} \times Se_R - an_{FNI} = Se_R \times (-an_{TPI} + an_{DP}) + (an_{TPI} - an_{DP}) \quad (S101)$$

Since $an_{DP} - an_{TPI} = an_{FNI}$, one can rewrite SM97 as:

$$an_{FNI} \times Se_R - an_{FNI} = Se_R \times an_{FNI} - an_{FNI} \quad (S102)$$

Which shows equality of the numerators. Therefore, the formulas are equal. Thus, I have shown equivalence of the Staquet [4] formulas and mine.

5.3 Comparisons to Prior Work: Conclusions

I have shown equivalence between my approach and the approaches of Gart and Buck [3] and Staquet et al.[4]

References

1. System of Linear Equations Calculator. Available online: https://www.emathhelp.net/calculators/algebra-2/system-of-linear-equations-calculator/ (accessed on 17 December 2022).
2. Youden, W.J. Index for rating diagnostic tests. *Cancer* **1950**, *3*, 32–35. https://doi.org/10.1002/1097-0142(1950)3:1<32::aid-cncr2820030106>3.0.co;2-3.
3. Gart, J.J.; Buck, A.A. Comparison of a screening test and a reference test in epidemiologic studies. II. A probabilistic model for the comparison of diagnostic tests. *Am. J. Epidemiol.* **1966**, *83*, 593–602. https://doi.org/10.1093/oxfordjournals.aje.a120610.
4. Staquet, M.; Rozencweig, M.; Lee, Y.J.; Muggia, F.M. Methodology for the assessment of new dichotomous diagnostic tests. *J. Chronic. Dis.* **1981**, *34*, 599–610. https://doi.org/10.1016/0021-9681(81)90059-x.
5. Expand Calculator. Available online: https://www.symbolab.com/solver/expand-calculator (accessed on 20 December 2022).
